# Squamoid Eccrine Ductal Carcinoma: Case Series of a Rare, Potentially Aggressive Skin Cancer Associated With Immunosuppression

**DOI:** 10.1111/cup.70127

**Published:** 2026-07-08

**Authors:** Xiang Li Tan, Pooja Patel, Jeva Cernova, Sarah Hogan, Jason Thomson, Efthymios Hadjimichael, Sharanpal Jeetle, Dorota Markiewicz, Michael Sheaff, Nilukshi Wijesuriya, Mark Hawthorne, Graeme Moir, Matthew Stodell, Kapil Bhargava, Suchitra Chinthapalli, Catherine A. Harwood

**Affiliations:** ^1^ Department of Dermatology Royal London Hospital, Barts Health NHS Trust London UK; ^2^ Department of Histopathology Royal London Hospital, Barts Health NHS Trust London UK; ^3^ Department of Histopathology Guys and St Thomas' NHS Foundation Trust London UK; ^4^ Department of Histopathology St George's University Hospital NHS Trust London UK; ^5^ Department of Plastic Surgery Royal London Hospital, Barts Health NHS Trust London UK; ^6^ Centre for Cell Biology and Cutaneous Research, Blizard Institute, Faculty of Medicine and Dentistry Queen Mary University of London London UK

**Keywords:** appendageal cancer, case series, organ transplant recipient, skin cancer, Squamoid eccrine ductal carcinoma

## Abstract

Squamoid Eccrine Ductal Carcinoma (SEDC) is a rare cutaneous adenexal carcinoma first described in 1997. It has distinct biphasic features on histology which commonly result in misdiagnosis as Bowen disease or cutaneous squamous cell carcinomas (cSCC) if diagnostic skin biopsies are too superficial. This may lead to a delay in diagnosis. It is locally aggressive with significant metastatic potential, and an association with immunosuppression has been previously reported in ~20% of cases. We conducted a single‐centre, retrospective cohort study of all patients diagnosed with SEDC over a 12‐year period (2013–2025). Ten cases were identified (7 M, 3 F; mean age at diagnosis 75 years [SD 10.8]), of whom 9 (90%) were immunocompromised due to solid organ transplantation (*n* = 6) or haematologic malignancy (*n* = 3). A diagnosis of SEDC was not initially made in any of the 4 cases in which an incisional biopsy was performed and only correctly diagnosed after complete excision. Locoregional recurrence occurred in 8 (80%) and distant metastasis in 2 (20%) of patients. Treatment included surgical excision in all cases, post‐operative radiotherapy due to incomplete or close margins or metastasis in 60%, and lymphadenectomy or parotidectomy in 20%. This case series highlights the diagnostic challenges posed by SEDC and the need for clinicopathologic vigilance, its potentially aggressive course, and the apparent strength of the association with immunosuppression which we propose may have been previously underestimated.

## 1 Introduction

Squamoid Eccrine Ductal Carcinoma (SEDC) is a rare cutaneous adnexal carcinoma first described in 1997 [1, 2], with 131 cases reported of which 27 are reported as immunosuppressed. It has distinct biphasic features on histology with both superficial squamous and deeper ductal eccrine differentiation commonly with positive staining for epithelial membrane antigen (EMA) and carcinoembryonic antigen (CEA) on immunohistochemistry highlighting the ducts. This biphasic pattern commonly results in misdiagnosis as Bowen disease or cutaneous squamous cell carcinomas (cSCC) if diagnostic skin biopsies are superficial, leading to delayed diagnosis. It is locally aggressive with significant metastatic potential [3, 4, 5, 6]. We report here a striking association with immunocompromise which has not previously been highlighted in the literature.

### 1.1 Case Reports

We conducted a single‐centre, retrospective cohort study of consecutive patients diagnosed with SEDC over a 12‐year period (2013–2025) in our institute. Clinical and histologic features were recorded and both treatment and clinical outcomes documented.

Ten cases were identified (7 M, 3 F) with a mean age at diagnosis of 75 years [SD 10.8] of whom 9 (90%) were immunocompromised due to solid organ transplantation (OTR (*n* = 6) or haematologic malignancy (myeloma, myelodysplasia, monoclonal gammopathy of undetermined significance, *n* = 3) (Table 1). Seven (70%) had a history of previous cSCC. Six (60%) were on immunosuppressive drugs including prednisolone (*n* = 3), ciclosporin (*n* = 3), mycophenolate mofetil (MMF) (*n* = 1), tacrolimus (*n* = 2), and rituximab (*n* = 1). Anatomic locations of SEDC included extremities in 50% (finger (*n* = 4; 40%); calf (*n* = 1)), head and neck region (*n* = 4), and upper chest (*n* = 1). Clinical features include scaly plaques and nodules, presented in Figure 1A–C.

**TABLE 1:** Clinical characteristics of the 10 cases of SEDC.

Case	Age (Y)	Sex	Site	Immune status	Previous keratinocyte cancers	Type of biopsy: diagnostic or excision	Diagnosis on initial biopsy	Duration of follow up	Local recurrence	Nodal recurrence	Distant metastasis	Disease associated death
1	63	F	Thumb	Kidney/liver transplant	Yes	Excision	SEDC	11Mo	Yes	Yes	Yes (Skin)	Yes
2	78	M	Ear	Kidney transplant	Yes	Incisional biopsy	Bowen disease	3Y^^^	No	Yes	No	No
3	69	M	Temple	Lung/kidneytransplant	Yes	Incisional biopsy	CSCC	1Y^^^	Yes	No	No	No
4	72	F	Calf	Kidney transplant	Yes	Excision	SEDC	2Y^^^	No	No	No	No
5	82	M	Thumb	Myelodysplasia MDS	No	Incisonal biopsy	CSCC or possible adenexal carcinoma with eccrine differentiation	4 Y	Yes	No	No	No
6	66	M	Forehead	Heart transplant due to dilated cardiomyopathy	Yes	Curettage and cautery	SEDC	5 Y	No	Yes	Yes (Bone and lung)	Yes
7	63	M	Middle finger	Kidney transplant × 2	Yes	Excision	SEDC	2 Y	Yes	No	No	No
8	79	M	Upper chest	No	Yes	Excision	SEDC	4Y	No	No	No	No
9	98	F	Little finger	MGUS	No	Incisional biopsy	CSCC	1 Y^^^	Yes	No	No	No
10	81	M	Parietal scalp	Myeloma	Yes	Excision	SEDC	1Y	Yes	Yes	No	No

Abbreviations: ^^^, transfer care to different hospital; +, positive margins/incomplete; AK, actinic keratosis; CsA, ciclosporin; CSCC, cutaneous squamous cell carcinoma; DoD, died of disease; F, female; Fr, fraction; FU, follow up; G, gray; KMnO_4_, Potassium permanganate; M, male; MGUS, monoclonal gammopathy of undetermined significance; MMF, Mycophenolate Mofetil; Mo, month; N, No; NA, data not available; PRED, prednisolone; RT, radiotherapy; SACT, systemic anti‐cancer therapy; SEDC, squamoid eccrine ductal carcinoma; TAC, tacrolimus; WLE, wide local excision; Y, year.

**FIGURE 1:**
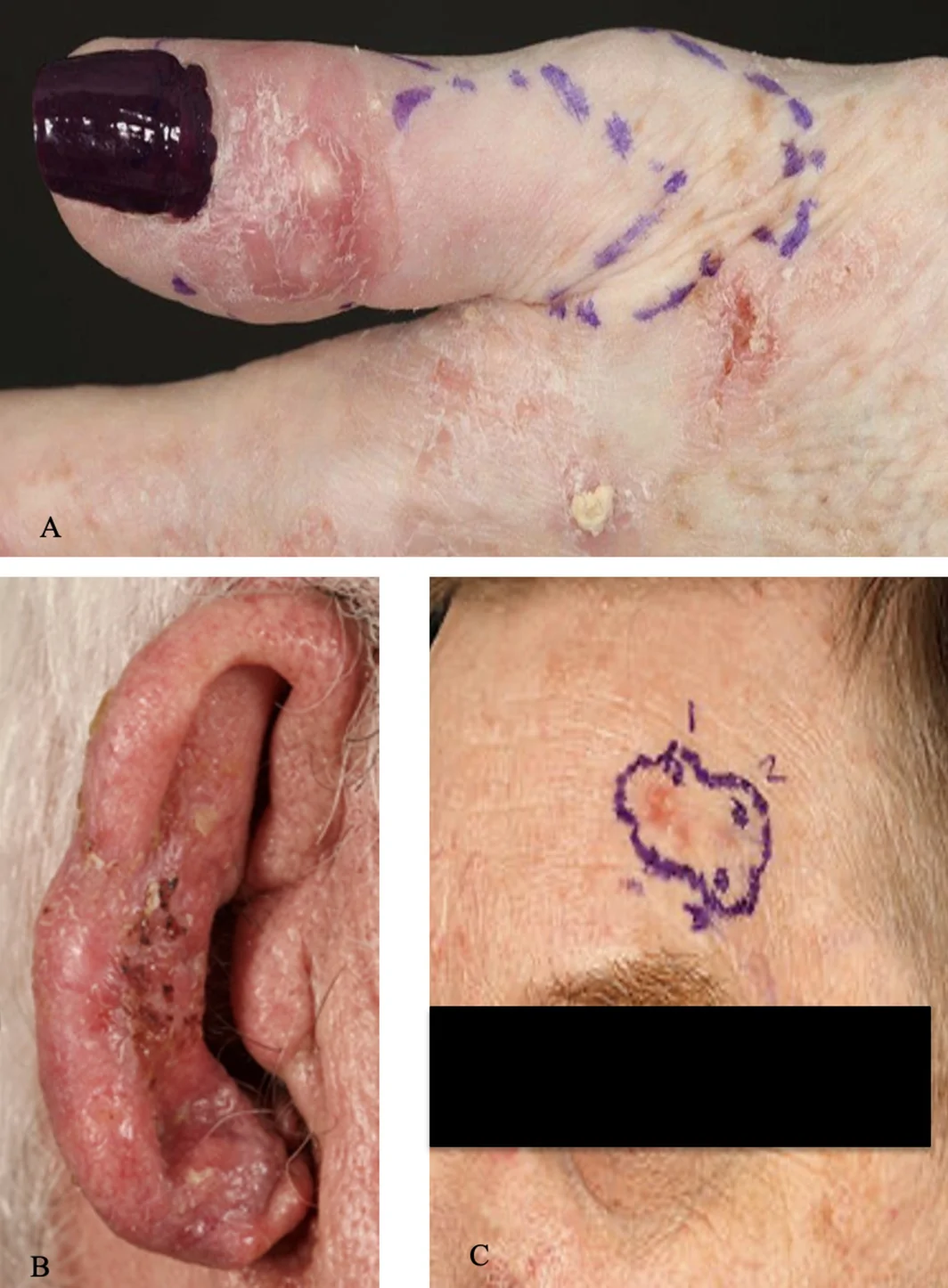
Squamoid Ductal Eccrine Carcinoma, clinical photographs. (A) Case 1: Initial lesion on thumb first identified in 2016. (B) Case 2: Initial lesion on the right helix in 2009. (C) Case 6: Initial scaly nodule on the right temple identified in 2013.

SEDC was diagnosed on initial histology in 6 cases (60%) in all of whom complete excision was undertaken. However, in the four patients in whom an initial diagnostic incisional biopsy was performed, a diagnosis of SEDC was not made and a diagnosis of cSCC (*n* = 3) or Bowen disease (*n* = 1) was the most common misdiagnosis. Histopathology and immunohistochemistry images are shown in Figure 2A,B. Tumors invaded up to the subcutis (Clark V) in 7 (70%), extending in to skeletal muscle in 3 (30%). Perineural invasion was identified in 50%, and lymphovascular invasion in 50%. Immunohistochemistry positivity included CEA, EMA (Figure 2C), cytokeratin (CK) 7, p40, GATA3, CK5/6, and BerEP4. The tumor frequently has connections to the epidermis with features of actinic keratosis or Bowen disease or well to moderately differentiated cSCC, with horn cysts, intercellular bridges, and squamous eddies. The deeper portion is characterized by eccrine ductal differentiation with an infiltrative growth pattern (Figure 2A,B). At higher power, these tumors exhibited nests, cords, or strands of epithelial cells with severe cytologic atypia and numerous mitoses within a desmoplastic stroma. The stroma may also be myxoid. The degree of differentiation varied, but well‐formed ducts were usually readily identifiable. The degree of cytologic atypia and number of mitoses (Figure 2A,B) per high power field tended to increase in the deeper portions of these tumors.

**FIGURE 2:**
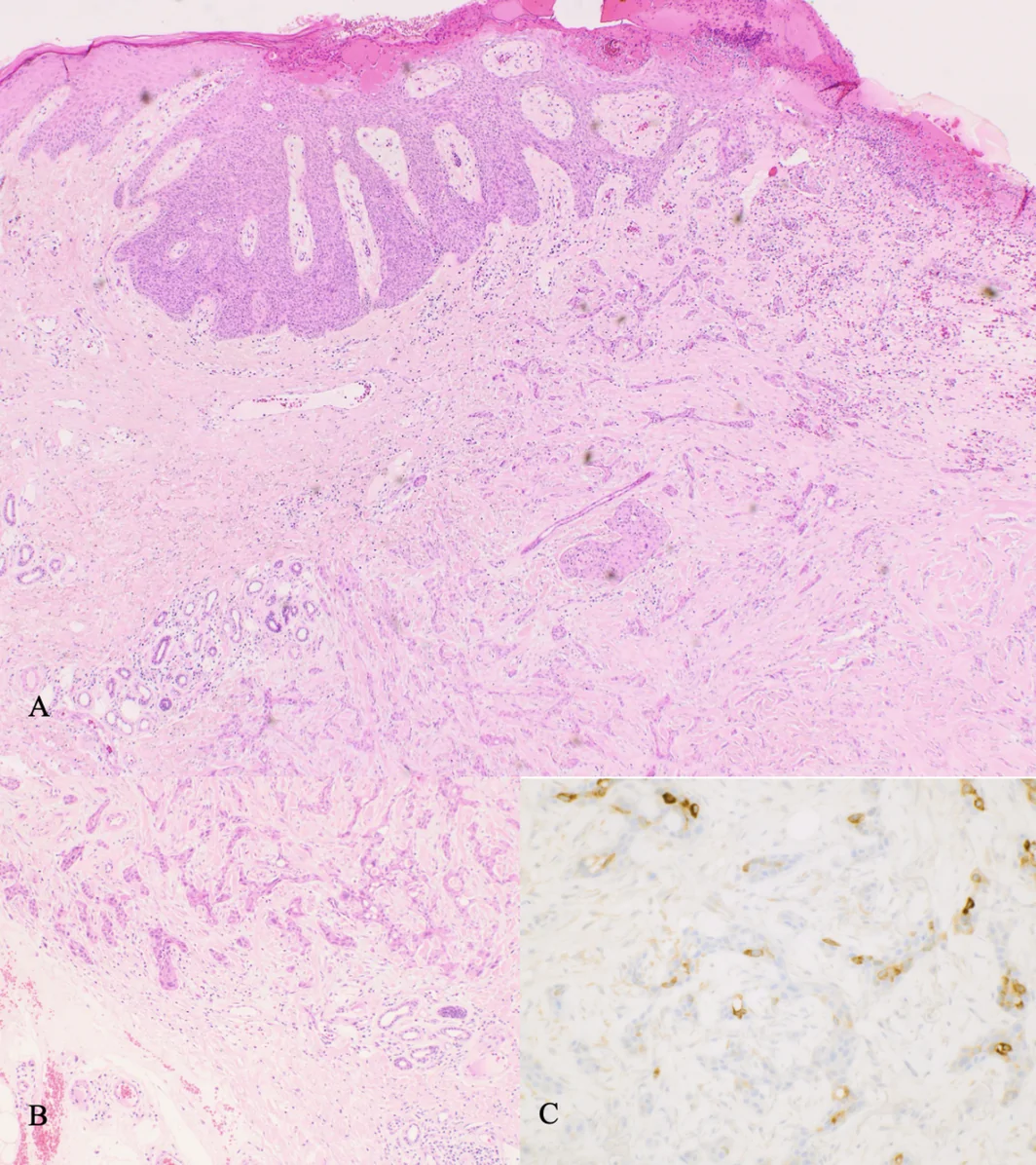
Squamoid Ductal Eccrine Carcinoma, histopathology slides. (A) Scanning power image depicting tumor zonation that is, nests of atypical cells superficially within the squamous zone above an area of dermal fibrosis, which is diagnostic of SEDC (H&E, 40×). (B) Higher power image demonstrating the infiltrative component arranged as nests and cords with ductal differentiation (H&E, 100×). (C) Immunohistochemistry staining positive for epithelial membrane antigen (EMA) (200×).

Treatment included wide local excision (WLE) in all cases, residual disease required further excision after this initial WLE. Post‐operative radiotherapy was performed in 60% (*n* = 6) due to incomplete or close margins despite re‐excision. The total follow‐up period ranged from 11 months to 5 years, with an average follow up of 30.6 months (SD 18.2). Locoregional recurrence (including local, in transit, nodal or intra‐parotid recurrence) occurred in 8 (80%) of cases (range: 4 months to 2 years). In 2 patients the initial diagnosis was cSCC and SEDC was only diagnosed after re‐excision of a local recurrence. In 3 patients in whom SEDC arose on a finger, amputation was required after surgery and radiotherapy. Four (40%) patients underwent lymphadenectomy and/or parotidectomy for nodal and/or parotid involvement. Two (20%) other patients with nodal disease were either managed with radiotherapy due to difficult site (ear) or switched to palliative care.

Distant metastases to the lungs and bones (*n* = 1), and distant skin metastasis (*n* = 1) occurred in 2 OTRs (20%) in whom previous surgery had been performed for nodal disease. Systemic therapy was felt not to be appropriate in either case after extensive multidisciplinary‐based and patient‐led discussions. In particular, both patients had multiple co‐morbidities impacting on the safety of systemic therapy, both had non‐kidney transplants (liver and cardiac, respectively), and therefore limited options in the case of graft rejection, and there is minimal evidence for the effectiveness of systemic therapy for metastatic SEDC. Both patients were therefore treated with palliative radiotherapy but died from metastatic SEDC (at 11 months and 5 years after initial diagnosis).

## 2 Discussion

This case series highlights the strength of the association between SEDC and immunosuppression. We also demonstrate the diagnostic challenges posed by SEDC, underscoring the need for clinicopathologic vigilance and the potentially poor outcomes in immunocompromised individuals.

Immunosuppression has previously been suggested as a risk factor for SEDC and a poor prognostic factor [4, 7, 8]. On review of the current literature, 27 of 131 (21%) reported cases of SEDC have presented in association with impaired immunity (Table 2). However, this may be a significant underestimate, as 59% of the cases in the literature did not comment on immune status. For example, this information is not provided in the largest published series of 30 cases [3]. This information is provided for all 10 of our patients and allows us to highlight the much stronger association with immunosuppression than previously reported, although we acknowledge that as a centre with both kidney transplant and haemato‐oncology services, there may be some referral bias in our patient population.

**TABLE 2:** Literature review of immunosuppressed SEDC cases.

Cases	Age (Y)	Sex	Clinical presentation/?clinical impression	Site	Duration before Dx	PMH/Immuno‐compromised	Treatment	Follow‐up (month)	Local recurrence	Nodal recurrence	Distant metastasis	Died of disease
Bellisario 2024 [9]	34	F	Erythematous papule with central fluctuance, peripheral induration? abscess	Thigh	4 weeks	Pregnant	1. I&D, Abx 2. WLE (complete)	Short FU	No	No	No	No
Clark 2013 [10]	75	M	Indurated plaque	L clavicle	5.5 M	CLL	MMS	12	No	No	No	No
Chan 2016 [11]	85	M	Ulcerated pruritic lesion	Scalp	6 M	Myasthenia gravis (AZA)	WLE	Short FU	No	No	No	No
Frouin 2015 [4]	74	F	Nodule? SCC	Nose	2 Y	KTR (15Y ago, CsA, corticosteroid) cSCC 11 Y ago	WLE (×5). Nose amputation Post op RT (65 Gy)	42	NA/YES^b^	NA/YES^b^	NA/YES^b^	Yes
Frouin 2015 [4]	71	F	Erythematous plaque with scales? Bowen disease, BCC, SSM	R Internal canthus	NA	Liver transplant 11 year before, Bowen disease, BCC, SCC	WLE (1 cm margin) Switch from CsA to tacrolimus	92.4	No	No	No	No
Frouin 2015 [4]	76	F	Nodule? BCC	R Cheek	1Y	Breast cancer	WLE	54	No	No	No	No
Fu 2009 [12]	75	M	?BCC	L Nasal wall	NA	MDS	MMS	3	No	No	No	No
Fu 2009 [12]	75	M	?BCC	L shoulder	NA	Pred for temporal arteritis, Waldenstrom macroglobulinemia	Initial Tx unknown MMS for recurent disease	66	No	Yes	No	No
Fu 2009 [12]	51	M	?Keratoacanthoma	R arm proximal	NA	HIV	MMS × 2, WLE superficial deltoid muscle, EBRT × 2, cetuximab	42	Yes	No	Yes	No
Fu 2009 [12]	55	M	?BCC	R shoulder	NA	HIV	MMS × 1	18	No	No	No	No
Fu 2009 [12]	55	M	Unknown initial clinical impression	R forehead	NA	KTR	MMS 2 3, WLE, EBRT × 2, cetuximab	48	No	Yes	No	No
Grube 2023 [13]	79	F	Crusted pink plaque, poorly demarcated	R forehead	NA	cSCC, polycythemia vera on ruxolitinib	MMS^a^ > plastics excision, skin graft, RT	Short FU	No	No	No	No
Herrero 1998 [14]	41	M	20 × 20 mm > 10 × 5 mm, a yellow fluid discharge	R knee	10 Y	Hodgkins lymphoma	WLE	Short FU	No	No	No	No
Honorato 2024 [7]	72	M	NA	Eyebrow	NA	KTR on tacrolimys, AZA, Pred	NA	LTF	No	No	No	NA
Honorato 2024 [7]	60	M	NA	Temporal region	NA	KTR on tacrolimus, everolimus	WLE (+intraoperative margin control by frozen sections)	36	Yes	No	No	No
Honorato 2024 [7]	62	M	NA	Scalp	NA	KTR on prednisolone	WLE (+intraoperative margin control by frozen sections)	22	Yes	Yes LN	Yes Lung	Yes
Jacob 2018 [15]	77	M	5‐cm, stellate, sclerotic plaque	L chest	2 Y	CLL, BCC, cSCC	WLE, RT	60	Yes	Yes	Yes	No
Lim 2022 [16]	78	M	Painless pink pearly papule with arborizing vessels	L lower lid‐cheek	4 M	cSCC, rectal cancer	1. MMS 2. Adjuvant RT for LVI	Short FU	No	No	No	No
Rovesti 2018 [17]	75	M	Nodular ulcerated lesion	R Temporal face	NA	Colon cancer, BPH	1. WLE X 3 (complete on 3rd) 2. MMS after recurrence	12	No	No	No	No
Svoboda 2021 [18]	83	M	1.5 × 1 cm	Scalp	NA	Crohns (AZA), melanoma	MMS	10	No	No	No	No
Svoboda 2021 [18]	82	M	2 × 2 cm	Scalp	NA	CLL	MMS	6.5	No	No	No	No
Svoboda 2021 [18]	81	F	4.7 × 4.5 cm	Scalp	NA	HIV, polymyositis (TAC, IVIg)	MMS	Short FU	No	No	No	No
Terushkin 2010 [19]	63	M	Solitary dermal nodule	L cheek	1Y	CLL	MMS	10	No	No	No	No
Harms 2024 [20]	52	M	NA	Chest	NA	Solid organ transplant	WLE	NA	No	No	No	No
Harms 2024 [20]	67	F	NA	Cheek	NA	Solid organ transplant	WLE	79.3	No	No	No	No
Harms 2024 [20]	94	M	NA	Ear	NA	CLL	WLE	13.0	No	No	No	No
Harms 2024 [20]	82	M	NA	Chest	NA	Solid organ transplant	WLE	51.8	No	No	No	No

Abbreviations: AZA, azathioprine; BCC, basal cell carcinoma; CLL, chronic lymphocytic leukemia; CSCC, cutaneous squamous cell carcinoma; G, gray; HIV, human immunodeficiency virus; I&D, incisioanl and drainage; IVIg, Intravenous Immunoglobulin; KTR, kidney transplant recipient; M, male; MMS, Mohs micrographic surgery; Mo, month; NA, not available; PRED, prednisolone; RT, radiotherapy; TAC, tacrolimus; WLE, wide local excision; Y, year.

^a^ Grube 2023: terminated due to bleeding and discomfort.

^b^ Frouin 2015: Assume locally advanced or metastatic as died of disease.

SEDC is commonly reported in elderly patients generally in the 7th and 8th decades on photodamaged skin, with head and neck, with face being the most common location [7]. Head and neck tumors occurred in 61%, followed by trunk 17%, extremities (upper and lower) 16%. However, in our case series, extremities accounted for 50% of sites, most commonly the fingers. In addition, clinical diagnosis may be challenging as clinical appearances may be subtle and usually present as small nodules or plaques. As in several of our patients, it can be misdiagnosed as cSCC or Bowen disease clinically as well as histologically, particularly in those with extensive field cancerization. SEDC has also been reported to simulate inflammatory or infective processes [21], also illustrated by the incorrect clinical diagnosis in our second patient, with consequent delayed diagnosis.

Adequate diagnostic biopsy is crucial as the features are indistinguishable from conventional squamous cell carcinoma if the ductal component (which is typically located in the deep dermis) is not represented in the sample and will miss the deeper glandular differentiation zone and duct‐like structures which are mostly located in the deep dermis. This limitation might account for the under‐diagnosis of squamoid eccrine ductal carcinoma. As in previously reported cases, initial biopsies have shown Bowen disease or cSCC. SEDC has a characteristic biphasic histology leading to tumor ‘zonation’ with squamous features predominating in superficial portions and syringomatous eccrine differentiation in the most invasive parts [4, 10, 22, 23, 24] The immunohistochemical profile of SEDC includes positivity for CEA, EMA and CK‐7 which highlight ductal differentiation and p63, CK5/6, 34beta E12, Cam 5.2, and MNF116 for the squamous portions. A differential diagnosis includes squamoid variant of ecrine porocarcinoma, which may look similar histologically; however, it lacks the zonation and cells look more ‘poroid’ with pre‐existing poroma.

Currently, there is no consensus on investigation, staging, management or follow up of SEDC. Our case series showed a higher local (60%) and regional recurrence rate (40%) than reported in the literature of immunocompromised (15% and 19%, respectively) or immunocompetent patients with no past medical history (PMH) reported (21% and 11%, respectively). This is proposed to be due to the diffusely infiltrative growth of the tumor with extension into the subcutis associated with perineural and lymphovascular invasion [2]. In our immunocompromised population, the rate of extension into the subcutis (70%), perineural (50%), and lymphovascular invasion (30%) was higher than reported by the largest case series (46%; 26%; 6%, respectively) [3]. Similarly, in a study reported by Frouin et al., in which 3 of 7 (43%) of patients were immunosuppressed, extension into the subcutis was reported in 5 of 7 (71%) of all tumors [4].

In addition to the locoregional recurrence risk, SEDC has potential for distant metastatic spread. Distant metastatic rates of 2% are reported in immunocompetent patients or patients with no PMH reported and 15% in immunosuppressed individuals. Our case series was similar to those previously reported in immunosuppressed patients with a rate of 20%, which suggests a higher risk of distant metastasis in immunosuppressed individuals. In addition, for organ transplant recipients, both in our cohort (*n* = 6) and in the literature (*n* = 9), SEDC‐associated mortality was 25% of the 8 cases with status reported [3, 4, 20]. The value of sentinel lymph node biopsy remains unknown; however, it may be considered in view of the risk for involvement of regional lymph nodes [3]. Positron emission tomography (PET) and computed tomography (CT) imaging have been proposed as staging investigations to exclude metastatic disease [5].

Mohs micrographic surgery (MMS) was conducted in 34 cases with 4 leading to locoregional recurrence (11.8%). Wide local excision (WLE) was conducted in 91 cases with 19 locoregional recurrences reported (21%). Post‐operative radiotherapy has been used in 18 cases of SEDC in the literature, of which 13 cases had no subsequent locoregional recurrence. Limited data are available on the role of adjuvant radiotherapy in SEDC although radiation has shown excellent locoregional control with acceptable toxicity for high‐risk adnexal neoplasms [25], evidence of its effectiveness is limited [2]. Of our 8 patients who received radiotherapy due to positive margins or metastasis, 2 required amputation despite radiotherapy, highlighting the aggressive nature of the tumor. Adjuvant radiotherapy in those with clear surgical margins may be considered if high‐risk features are present, including perineural invasion, multifocal and recurrent disease, but further studies are required [25]. SEDC treated with multimodal approaches may still lead to late recurrences, necessitating long‐term surveillance. Median time to recurrence has been reported to be 14 to 24 months [2, 3, 4]. No guidelines are available regarding surveillance for locoregional recurrences and metastatic disease. Wide local excision, post‐operative adjuvant radiotherapy and follow‐up imaging surveillance may be justified; however, larger studies will be required to better inform evidence‐based management recommendations.

In conclusion, we show the close association between SEDC and immunocompromise with higher rates of locoregional recurrence and distant metastasis. We also highlight the diagnostic challenges and its aggressive nature, potentially pointing to the need for a multimodal treatment approach. Further studies are required to establish treatment guidelines for this rare aggressive adnexal tumor. Increased recognition and reporting of this rare malignancy will improve understanding of its true incidence, metastatic potential, and optimal management approaches.

## Funding

The authors have no relevant funding to report.

## Conflicts of Interest

The authors declare no conflicts of interest.

## Data Availability

The data that support the findings of this study are available from the corresponding author upon reasonable request.
